# A New Multilocus Sequence Typing Scheme and Its Application for the Characterization of *Photobacterium damselae* subsp. *damselae* Associated with Mortality in Cetaceans

**DOI:** 10.3389/fmicb.2016.01656

**Published:** 2016-10-21

**Authors:** Patricia Alba, Andrea Caprioli, Cristiano Cocumelli, Angela Ianzano, Valentina Donati, Francesco Scholl, Luigi Sorbara, Giuliana Terracciano, Gianluca Fichi, Fabio Di Nocera, Alessia Franco, Antonio Battisti

**Affiliations:** ^1^General Diagnostic Department, Sede Centrale, Istituto Zooprofilattico Sperimentale del Lazio e della ToscanaRome, Italy; ^2^Pathology Department, Sede Centrale, Istituto Zooprofilattico Sperimentale del Lazio e della ToscanaRome, Italy; ^3^Sezione di Pisa, Istituto Zooprofilattico Sperimentale del Lazio e della ToscanaPisa, Italy; ^4^Animal Health Department, Istituto Zooprofilattico Sperimentale del MezzogiornoPortici, Italy

**Keywords:** *Photobacterium damselae*, MLST, genotyping, phylogeny, cetaceans, infection, zoonoses

## Abstract

*Photobacterium damselae* subsp. *damselae* (PDD) is a known pathogen of fish, humans and marine mammals. In this study, a Multilocus Sequence Typing (MLST) scheme based on six housekeeping genes (g*lp, gyrB, metG, pnt, pyrC*, and *toxR*) was developed to better understand the PDD population structure and used to type 73 PDD isolates from cetaceans, mainly striped dolphins (*Stenella coeruleoalba*) involved in mortality episodes, and from a few marine chelonians. Five reference ATCC strains were also included in the study. Typing allowed the discrimination of groups of PDD strains isolated from different host species, at different times and from different geographic areas, suggesting that a clonal PDD group may have spread in the Tyrrhenian sea at the time of an Unusual Mortality Event (UME) among cetaceans, mainly striped dolphins, occurred in early 2013 along the Italian western coasts.

## Introduction

*Photobacterium damselae* subsp. *damselae* (PDD) is a marine Gram-negative bacterium belonging to the *Vibronaceae* family, classified in the same species as *Photobacterium damselae* subsp. *piscicida* (PDP), the causative agent of pasteurellosis in fish (Andreoni and Magnani, 2014). As other members of the *Vibrionaceae* family, PDD possess two chromosomes (Okada et al., 2005) and harbor at least two large plasmids (Rivas et al., 2011; Nonaka et al., 2012). Both subspecies share more than 99% of the 16S DNA sequence and show a percentage of DNA-DNA hybridisation greater than 80% (Gauthier et al., 1995). Despite their genetic similarity, the two subspecies can be easily distinguished by phenotypic characteristics. Differential phenotypic tests, only positive for the subsp. *damselae* include growth at 37⋅C, hemolytic activity on sheep blood agar, motility, nitrate reduction, and production of urease and amylase (Thyssen et al., 1998; Rivas et al., 2013a).

PDD is an autochthonous member of aquatic ecosystems, but it is also considered a primary pathogen of a variety of marine animal species, including crustaceans, fish, and molluscs (Rivas et al., 2013a). In humans, PDD is considered a zoonotic pathogen (Austin, 2010) and can cause opportunistic infections frequently originating from wounds exposed to salt or brackish waters and inflicted during marine activities (Takahashi et al., 2008), that may evolve into necrotizing fasciitis leading to a fatal outcome (Rivas et al., 2013a). In marine mammals, PDD was reported for the first time as a dolphin pathogen in 1988, when it was isolated from the wound of a bottlenose dolphin (*Tursiops truncatus*; Fujioka et al., 1988). Later on, it was also isolated from healthy and stranded dolphins in the USA and along the Mexican coasts (Buck et al., 1991, 2006), and from other cetaceans, such as the Bryde's whale (*Balaenoptera brydei*; Buck et al., 1991).

In the Mediterranean Sea, several dolphin mass mortality episodes have been described in the past 25 years, essentially attributed to dolphin morbillivirus infections (Casalone et al., 2014). In 2013, PPD was isolated from many cetaceans, mainly striped dolphins (*Stenella coeruleoalba)*, involved in an Unusual Mortality Event (UME) occurred along the Tyrrhenian Sea coast of Italy (Casalone et al., 2014). Together with dolphin morbillivirus, other infectious agents, and environmental factors, it was considered among the possible determinants involved in the event, even though no definitive conclusions could be drawn (Casalone et al., 2014).

PDD pathogenicity has been related to its capability of producing different virulence proteins, such as haemolysins and histamine (Chiu et al., 2013), and to the presence of an iron uptake system (Rivas et al., 2013b). In particular, it has been reported that haemolytic strains produce a chromosome-encoded haemolysin, termed HlyA_*ch*_, while highly haemolytic strains also harbor the virulence plasmid pPHDD1, which encodes two other different haemolysins: damselysin (Dly) and HlyA_*pl*_ (Rivas et al., 2013b), recently renamed phobalysin (PhlyP) (Rivas et al., 2015).

Different molecular techniques have been used for PDD genotyping, including Amplified Fragment Length Polymorphism (AFLP) (Botella et al., 2002; Takahashi et al., 2008), Randomly Amplified Polymorphic DNA (RAPD) (Labella et al., 2010; Chiu et al., 2013; Khouadja et al., 2014), Pulsed Field Gel Electrophoresis (PFGE) (Takahashi et al., 2008; Chiu et al., 2013), Ribotyping (Takahashi et al., 2008), Multilocus Sequence Typing (MLST) (Takahashi et al., 2008), Enterobacterial Repetitive Intergenic Consensus (ERIC)-PCR fingerprinting (Labella et al., 2010), and Repetitive Element Palindromic PCR (REP-PCR) (Labella et al., 2010). However, all these techniques have underlined high genetic intraspecific variability, making it difficult to understand the genetic relationship among isolates and to describe the PDD population structure, often lacking congruence with phenotypic results. MLST is currently considered the method of choice when describing the population structure of several bacterial species, for reconstructing phylogeographic relationships among isolates, and for clinical epidemiology purposes. An advantage of the MLST approach is that this tool has proven to be a robust and portable method for the genetic characterization of the isolates of a bacterial population, since it allows the comparison of the results between laboratories (Maiden, 2006; Glaeser and Kämpfer, 2015). Among the different typing methods, a MLST scheme using three theoretical housekeeping genes (*gyrB, toxR*, and *ompU*), previously reported to be suitable for phylogenetic studies of *Vibrio* species, has been already used to study clinical and environmental PDD isolated in Japan (Takahashi et al., 2008) although, according to the authors themselves, this method presented limitations.

The aim of this study was to propose an improved MLST scheme for PDD genotyping. Using this scheme, we investigated the genetic structure of PDD isolates mainly collected from cetaceans during passive surveillance activities of mortality episodes conducted in Italy in recent years, either from sporadic events or clusters of cases occurred within a short period of time. Isolates were further characterized by determining some of their virulence genetic and phenotypic characteristics. A collection of reference strains was also included in the study.

## Materials and methods

### Bacterial isolates

A total of 78 *Photobacterium damselae* subsp. isolates were analysed (Supplementary Table 1). PDD isolates were collected between 2012 and 2014 from the following species stranded along the Tyrrhenian coasts of Italy: 42 cetaceans including 31 striped dolphins (*S. coeruleoalba*), 5 bottlenose dolphins (*T. truncatus*), 3 sperm whales (*Physeter macrocephalus*), 1 short-beaked common dolphin (*Delphinus delphis*), and 2 chelonians (loggerhead sea turtle, *Caretta caretta*). More isolates from a same animal were included in the study only when detected from different organs, or when presenting a different haemolytic phenotype. Out of the 78 isolates, 42 were from striped dolphins stranded during a short period of time (January–March 2013) and reported as a cluster of death cases, defined as UME (Casalone et al., 2014). Four PDD strains from the ATCC collection (ATCC 33539, ATCC 35083, ATCC 51805, and ATCC 51807) and one PDP strain (ATCC 51736), all isolated from different saltwater fish species, were also included.

Isolates were cultured in 5% sheep blood agar (bioMérieux, Craponne, France) and incubated at 37⋅C for 24 h. Identification at species and subspecies level was obtained by assessing the phenotypic characteristic (including the capacity to grow at 37⋅C, the haemolytic activity on sheep blood agar and the motility), following microscopy examination, and biochemically by using the API 20E identification system (bioMérieux, Craponne, France), in which urease activity differentiated PDD from the urease-negative PDP.

### Multilocus sequence typing (MLST)

A MLST scheme was developed choosing six PDD housekeeping genes: gyrB (DNA gyrase, subunit B) and toxR (positive transcriptional regulator-ToxR protein), both already proposed by Takahashi et al. (2008), glp (Glucose-6-phosphate isomerase), metG (Methionyl-tRNA synthetase), pnt (Transhydrogenase alpha subunit), and pyrC (Dihydroorotase). These latter were selected as they had been previously used for the *Vibrio vulnificus* MLST scheme (Bisharat et al., 2007; http://pubmlst.org/vvulnificus/). All chosen genes belonged to the PDD chromosome I, except for pnt, belonging to the chromosome II.

Following DNA extraction, PCR reactions were performed using the following thermal conditions: 10′ at 95⋅C, followed by 35 amplification cycles with conditions as reported in the Supplementary Table 2. Every mix reaction contained 0.2 mM of dNTP's, 0.5 μM of each primer, 1.5 mM of Mg_2_Cl and 2U of taqPolymerase Gold (Applied Biosystem, Foster City, CA, USA). Primers were designed with the CLC DNA workbench® software version 5.7.1 (CLC Bio-Qiagen, Aarhus, Denmark) using the PDD (ADBS01000000) as reference genome. Amplicons were Sanger sequenced by BigDye Terminator chemistry (Applied Biosystems) using the same primers. Sequence data analysis and classification was performed using the CLC DNA workbench® software version 5.7.1 (CLC Bio-Qiagen).

A different number was assigned to each allele of the six genes studied. In the same way, a different sequence type (ST) was assigned to each combination of alleles. The ratio between non-synonymous substitutions and synonymous substitutions (dN/dS) (Nei and Gojobori, 1986) and the index of association (Smith et al., 1993) was calculated using START2 (Jolley et al., 2001). The Tajima's D neutrality test (Tajima, 1989), the Fu and Li's F neutrality test (Fu and Li, 1993), the number of variable sites, the number of point and InDel mutations and the average of guanine plus cytosine (G+C) for the proposed genes were calculated using DnaSP v.5.0 (Librado and Rozas, 2009). Clonal complex (CC) assignment was performed using eBURSTv.3 software (http://eburst.mlst.net/; Feil and Enright, 2004), and confirmed with the graphical representation of the Minimum Spanning Tree (MST). The MST was constructed with the Bionumerics® 7 software (Applied Maths, Sint-Martens-Latem, Belgium), using the allele combinations as categorical values. The ClonalFrame software, with default parameters, was used to assess the amount of recombination among the isolates under study and among the obtained STs, by calculating the r/m ratio (ratio of rates at which nucleotides become substituted as a result of recombination and mutation; Didelot and Falush, 2007). This ratio is usually categorized as low (< 1), intermediate (1–2), or high (>2) (Vos and Didelot, 2009).

### Multilocus sequence analysis (MLSA) and phylogenetic analysis

The 78 sequences of each gene were aligned using Clustal IW with MEGA6 (Tamura et al., 2013), translated to aminoacids and back-translated to nucleotides. *ToxR* was previously aligned using clustal Omega (http://www.ebi.ac.uk/Tools/msa/clustalo/) in order to mark off the InDel gaps. For each gene (*glp, gyrB, metG, pnt, pyrC* and *toxR*) the evolutionary history (MEGA6) was inferred using the Maximum Likelihood (ML) method (Tamura-Nei model; Tamura and Nei, 1993) and rooted with the PDP sequence of each gene. Initial trees for the heuristic search were automatically obtained by applying Neighbor-Joining (NJ) and BioNJ algorithms to a matrix of pairwise distances estimated using the Maximum Composite Likelihood (MCL) approach, and then selecting the topology with superior log likelihood value. All positions containing gaps and missing data were eliminated. In the final dataset a total of 480 (*glp*), 537 (*gyrB*), 429 (*metG*), 396 (*pnt*), 507 (*pyrC*), and 372 (*toxR*) positions were present, respectively.

The sequences obtained from the 78 isolates were concatenated for each isolate in the following order using START2 (Jolley et al., 2001): *glp, gyrB, metG, pnt, pyrC*, and *toxR*. For comparison purposes, the phylogenetic tree was built using: (a) the Neighbor-joining method based on a multiple alignment (with Kimura corrections), and with the Jukes and Cantor correction, using the Bionumerics® 7 software. None position was discarded, for a total of 2739 bases in the final dataset. The tree topology was tested using 500 bootstrap replicates; (b) the Minimum Evolution method (Rzhetsky and Nei, 1992), using MEGA6 (Tamura et al., 2013), with the Neighbor-joining algorithm (Saitou and Nei, 1987) used to generate the initial tree; (c) a Bayesian approximation using Markov chain Monte Carlo (MCMC) algorithms with the BEAST software v1.8 (Drummond et al., 2012).

### Haemolytic phenotype of the PDD isolates

The haemolytic activity of the PDD isolates was classified on the basis of the diameter of the haemolytic halo observed on 5% sheep blood agar plates (bioMérieux, Craponne, France), following overnight incubation of a single colony, as previously described (Rivas et al., 2013b). Isolates were classified into four distinct groups based on the haemolytic phenotypes: large haemolysis (LH, halo diameter ≥ 7 mm), medium haemolysis (MH, halo diameter from 5 to 7 mm), small haemolysis (SH, halo diameter from 2 to 4 mm) and no-haemolysis (NH).

### Haemolysin genes detection

The presence of the following haemolysin coding genes was investigated: d*ly, hlyA*_pl_, and h*lyA*_cr_. Genomic DNA was extracted as above described. Genes were tested using simplex PCRs according to previous protocols (Osorio et al., 2000; Rivas et al., 2014). Amplicons of the *dly* gene were confirmed by sequencing with the same primers and compared with the reference sequence FN597600.2 using BLAST (http://www.ncbi.nlm.nih.gov/BLAST/).

### Plasmid detection

Seventeen PDD isolates were selected for plasmid detection on the basis of the time of detection (i.e., UME vs. non UME), haemolysis phenotype and its genetic basis (Table 1). Plasmids were detected by using a S1 nuclease-PFGE with the following in-house protocol. PDD isolates grown on sheep blood agar plates (bioMérieux, Craponne, France) were resuspended in a 2 ml Cell Suspension Buffer (10 mM Tris-HCl; 1 mM EDTA; pH 8.0) until reaching a turbidity of 5 McFarland. Two hundreds μl of this suspension were treated with 10 μl of proteinase K (20 mg/ml) and mixed with 100 μl of a 2% melted agarose in TE buffer. After solidification, the obtained plug was incubated for 2 h at 55⋅C in 1 ml Cell Lysis Buffer (50 mM Tris-Hcl; 50 mM EDTA; 1% sarcosyl; pH 8.0) with 5 μl of proteinase K (20 mg/ml). A 3 mm slice of the plug was restricted with 5 U of S1 nuclease (Promega, Madison, USA) for 45′ at 37⋅C. Electrophoresis was performed in a CHEF-II (Bio-Rad Laboratories GmbH, Munich, Germany) at 6 V/Cm with an initial pulse of 2.2 and a final pulse of 63.8 for 22 h. *Salmonella enterica* subsp. *enterica* serotype Braenderup H9812, restricted with XbaI (Promega) for 2 h at 37⋅C was used as size marker (Hunter et al., 2005).

**Table 1:** **Description of the 17 isolates selected for plasmid detection and subjected to S1 nuclease-PFGE**.

ID^*^	**Source of isolation**	Haemolytic phenotype^*^	**Detected haemolysin genes**	**Number of plasmids detected**	**Approximate plasmid size (Kb)**
13011826	Spleen	LH	*dly*	1	73
13011826	Brain	LH	*dly*	4	121; 99; 43; 30
13011852	Brain	LH	–	3	69; 40; 32
13011852	Spleen	LH	*dly*	3	112; 50; 43
13011852	Lymph node	MH	*dly; hly_cr; hly_pl*	1	148
13009643	Liver	MH	*dly; hly_cr; hly_pl*	2	184; 75
13017376	Peritoneal fluid	MH	*hly_cr*	2	51; 32
13017376	Brain	MH	*hly_cr*	1	49
13011852	Intracardiac clot	NH	–	1	49
13011960	Brain	NH	–	2	55; 33
13017376	Peritoneal fluid	NH	–	n.d.	n.a.
13017376	Brain	NH	–	1	31
13072252 A	Intracardiac clot	MH	*hly_cr*	n.d.	n.a.
13072252 B	Intracardiac clot	SH	–	4	107; 73; 59; 51
13058002	Brain	SH	*hly_cr*	n.d.	n.a.
13055678	Lymph node	NH	–	n.d.	n.a.
ATCC 33539	ATCC	MH	*dly; hly_cr; hly_pl*	2	307; 51

*n.d., not detected; n.a., not applicable*.

* *LH, large haemolysis; MH, medium haemolysis; SH, small haemolysis; NH, no-haemolysis*.

* *Each number identify a single animal*.

### Statistical analysis

The data are presented as absolute frequencies and percentages (%). In order to determine whether the association and the magnitude of discrepancy between two proportions was significant (e.g., presence of a certain CC and haemolytic activity of the isolates collected during UME or non-UME periods), the chi-square test was applied. Odds Ratio and a 95% confidence interval (CI) were estimated by using the StatCalc utility of the Epi Info™ version 7.1.5 software (http://wwwn.cdc.gov/Epiinfo/7/index.htm). A *p* < 0.05 was considered statistically significant.

## Results

### Multilocus sequence typing (MLST)

Twenty-one different haplotypes were observed for *glp*, 24 for *gyrB*, 21 for *metG*, 17 for *pnt*, 25 for *pyrC*, and 29 for *toxR*, the latter being the only one presenting insertions/deletions (InDel) mutations (Table 2). In all cases, the dN/dS ratio value for the used genes was < 1. The Tajima's D, and Fu and Li's *F*-tests were not statistically significant (*p* > 0.05; Table 2).

**Table 2:** **Genes used in the MLST scheme, including general description and statistical analysis**.

**Gene**	**Fragment length**	**% G+C**	**Haplotypes**	**Number of**	**Total number**	**dN/dS**	Tajima's D^*^	**Fu and Li's F**	**InDel**
	**analysis (bp)**		**Haplotypes**	**variable sites**	**of mutations**				**mutations**
*glp*	480	46.5	21	25	25	0.0513	−1.66632^¥^	−2.53677^¥^	n.a.
*gyrB*	537	44	24	28	28	0.0433	−0.59551^§^	−1.28524^§^	n.a.
*metG*	429	42.4	21	20	20	0.0169	−1.56838^§^	−2.35963^¥^	n.a.
*pntA*	396	46.1	17	20	20	0.0050	0.85313^§^	0.3387^§^	n.a.
*pyrC*	507	42.6	25	40	44	0.0591	−0.18696^§^	−0.51942^§^	n.a.
*toxR*	372–390^*^	42.7	29	40	44	0.1064	0.49313^§^	0.14047^§^	4 haplotypes,
									affecting 18 sites

* *For the statistical analysis, the gaps were excluded*.

¥ *Statistical significance: Not significant, 0.10> P > 0.05*.

§ *Statistical significance: Not significant, P > 0.10. n.a. = not applicable*.

**Figure 1:**
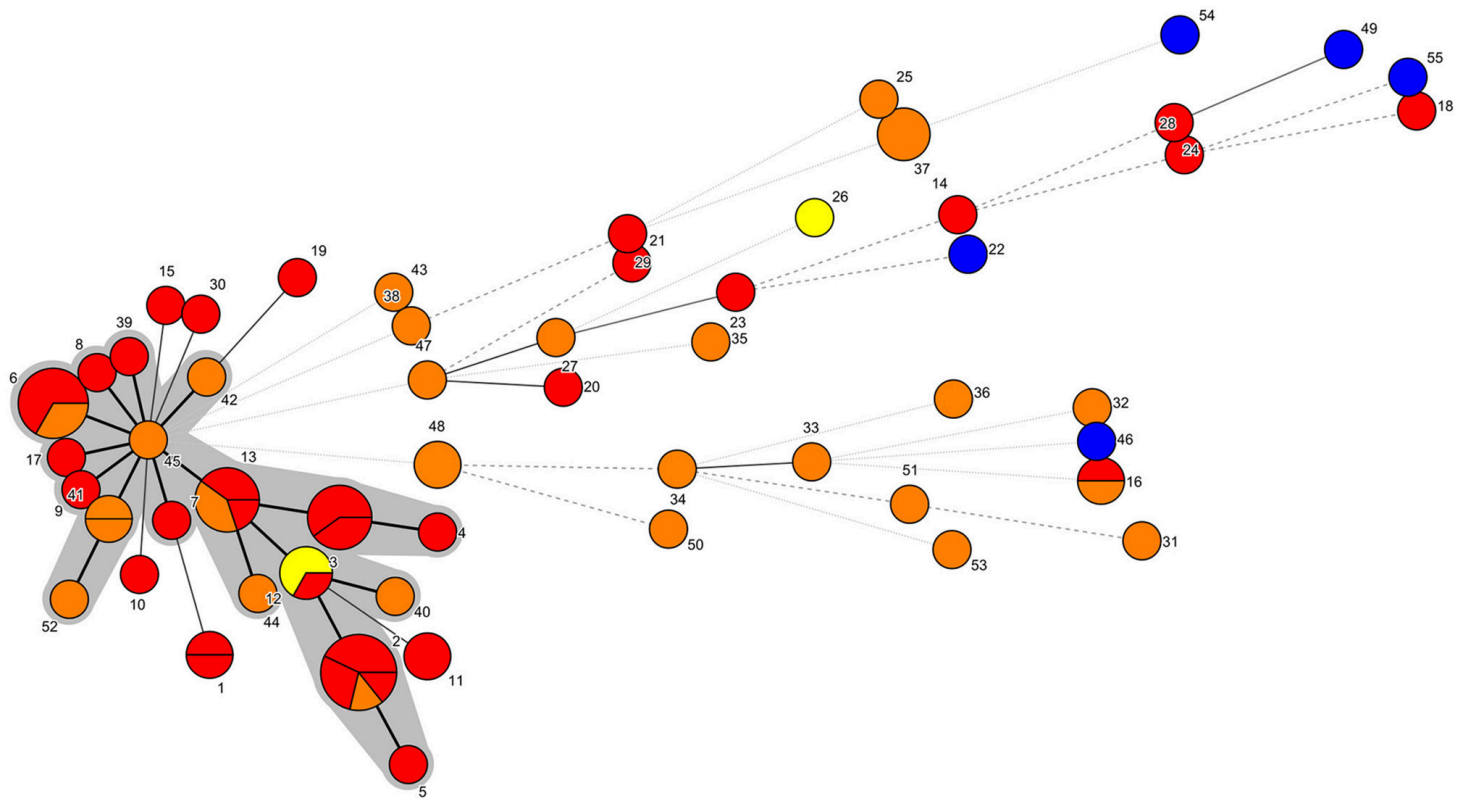
**MST representation**. Each node represents a different ST, which is indicated by a number. The number of partitions represents the number of isolates belonging to that ST. Bold continuous lines link isolates with STs which differ in one allele only. Light continuous lines link isolates with STs differing in two alleles. Dotted lines (bold or light) link isolates whose STs presented three or more alleles of difference. The shaded area includes the isolates belonging to the CC45. Yellow: PDD isolated before January 2013. Red: PDD isolated between January 2013 and March 2013. Orange: PDD isolated after March 2013. Blue: ATCC PDD and PDP strains.

The combination of the different alleles defined 55 different STs. ST2, with seven isolates, and ST6, with five isolates, were the most frequent. Using the eBURST analysis, 18 STs comprising a total of 36 isolates were subsequently grouped into a major CC, named CC45. On the other hand, 42 isolates were classified in 37 singleton STs. These results were in agreement with the MST result (Figure 1).

As for the Maynard Smith Index of Association (I_A_), a 2.0774 value was obtained, with an observed variance greater than the expected variance obtained in 1000 trials (*p* < 0.001), showing a linkage disequilibrium within the studied population.

As for the recombination amount, the r/m ratios obtained were r/m = 1.13 (1.1–1.16) for all the isolates, and r/m = 1.96 (1.88–2.04) for the 55 different STs.

The proposed MLST PDD database, with the sequences associated for each allele, is available at http://pubmlst.org/pdamselae/ (Jolley and Maiden, 2010).

### Phylogenetic analysis

The phylogenetic trees of the *pyrC, pnt*, and *toxR* genes, constructed using the Maximum Likelihood method, showed the presence of a main group of isolates presenting a high genetic relatedness. The analysis of the *glp, gyrB*, and *metG* genes helped to discriminate isolates that appeared to be evolutionarily more distant, probably since these genes seems to be more stable over time (Supplementary Figures 1–6). The PDP isolate showed a specific combination of unique alleles, except for the allele six of the *metG* gene, that was also present in three PDD isolates (Supplementary Table 1).

**Figure 2:**
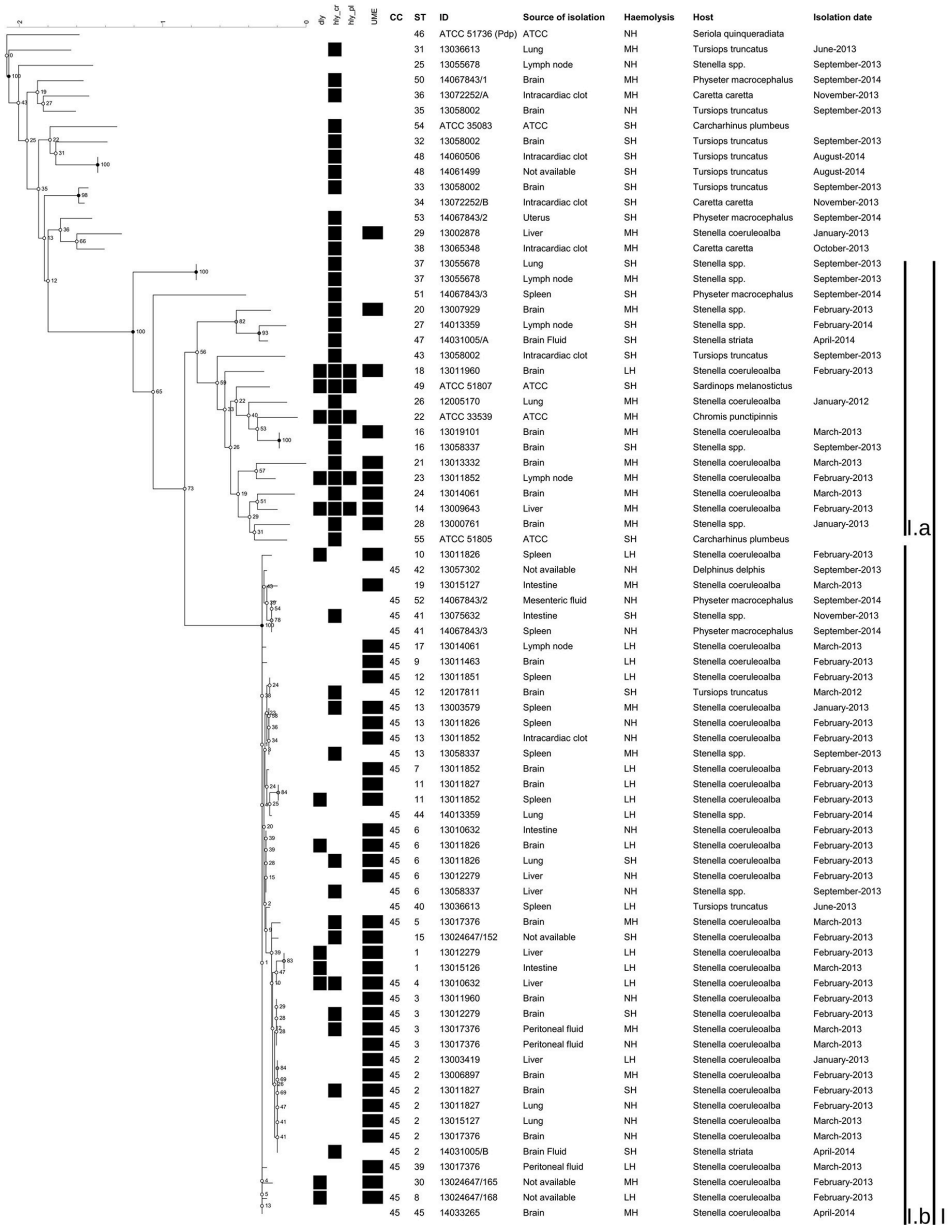
**Phylogenetic tree of the concatenated sequences built using Bionumerics**. Phylogenetic tree constructed using Neighbor-joining method based on a multiple alignment. None position was discarded, having a total of 2739 bases in the final dataset. The tree topology was tested using 500 bootstrap. The haemolysin genes detected, the defined UME group, the CC, the ST, the haemolytic phenotype, the dates of isolation, the ID of the isolates, the sources of isolation and the hosts were included.

The NJ phylogenetic tree of the concatenated sequences allowed the separation of a main large cluster (I), which contained all the isolates retrieved during the UME period but one, with a bootstrap value of 100. This main cluster also contained a subcluster (I.b), that grouped the isolates exhibiting the highest homology level and included all the CC45 isolates and related STs (Figures 1, 2). Moreover, the majority (33/44, 75%) of the isolates included in the I.b subcluster were from cetaceans stranded during the UME period. Another subcluster (I.a), with a lower (56) bootstrap value consisted of more genetically different isolates, including the three ATCC PDD strains.

The phylogenetic trees obtained with the Minimum Evolution method and with the Bayesian approximation method resulted very similar to the NJ output, and are presented as Supplementary Figures 7, 8, respectively.

### Haemolytic phenotype of the PDD isolates

Of the 73 field isolates, 17 were classified as LH, 22 as MH, 19 as SH, and 15 as NH. Three ATCC strains were classified as SH, and one as MH. The PDP strain was classified as NH (Supplementary Table 1).

In total, 39 out of 73 (53.4%) field PDD isolates presented the LH or MH phenotypes, and 29 of them (74.4%) were from cetaceans stranded during the UME period. The rest of the PDD field isolates (34/73, 46.5%) presented the SH/NH phenotypes. Thirteen of these strains (38.23%) were isolated during the UME period (Supplementary Table 1).

### Haemolysin genes detection

Overall, 50 out of the 78 investigated isolates (64.1%) were positive to at least one of the haemolysin genes tested, with a variety of combinations (Supplementary Table 1). In particular, 13 (16.5%) isolates tested positive for the *dly* gene. Of these, eight had the LH phenotype, four the MH phenotype and one the SH phenotype. The *hly_pl_* gene was detected only in five out of 78 isolates tested (6.5%). Of these, one had the LH phenotype, three the MH phenotype and one the SH phenotype. The *hly*_cr_ gene was detected in 43 out of 78 isolates tested (55%). Of these, two had the LH phenotype, 19 the MH phenotype, 21 the SH phenotype, and one the NH phenotype.

As a whole, only five of the 78 isolates (6.4%) were positive for all the three considered genes: one LH field isolate from the brain of a striped dolphins, two MH field isolates from the liver and lymph nodes of two striped dolphins, the PDD ATCC 33539 MH strain, and the PDD ATCC 51807 SH strain (Supplementary Table 1). Nine LH isolates (9/17, 50.3%) were negative for all the three genes, as well as three MH isolates (3/23, 13%), one SH isolate (1/22, 4.5%), and 15 NH isolates (15/16, 93.8%).

The *dly* amplification products of three LH isolates were sequenced and compared with the deposited sequence of the pPHDD1. Two of them (ID: 13011826 LH spleen and 13011852 LH spleen) showed a 100% similarity (540 bp) with the deposited *dly* sequence (FN597600.2). The other sequence (ID: 13010632 LH liver) had only a 90% similarity (540 bp) with the reference *dly*, differentiating for 20 non-synonymous mutations, one deletion and 32 synonymous mutations. The three sequences obtained were deposited in the GenBank nucleotide collection with the following accession numbers (KX589487, KX589488, and KX589489), respectively.

### Plasmid detection

The S1 nuclease-PFGE of the 17 selected isolates showed that only one MH *dly*+, *hly*_*pl*_+, and *hly*_*cr*_+ isolate presented a plasmid of the approximate size of the pPHDD1 plasmid (150 kb). The other LH, SH and NH isolates tested presented a variable number of different plasmids, from none to 4, with size ranging from about 30 to 300 kb (Table 1).

### Statistical analysis

The association between proportions of PDD isolates categorized by CC (CC45 vs. non-CC45), or by the haemolytic phenotype (e.g., LH+MH vs. SH+NH) and isolation during UME and non-UME periods were statistically significant (Chi-square *p* < 0.05 and *p* < 0.01, respectively). The Odds Ratios were 2.67 (95% CI 1.02–6.98) and 4.68 (95% CI 1.72–12.70), respectively (Supplementary Table 3).

## Discussion

In the present study we provided an improved MLST scheme for the genotyping of PDD, a marine bacterial species with a complex genome. MLST is still one of the most frequently used sequence-based genotyping methods, highly unambiguous, reproducible and portable (Maiden, 2006). The proposed MLST is composed of five housekeeping genes belonging to the PDD chromosome I, and one, pnt, belonging to the chromosome II. Despite the toxR gene presented insertion/deletion mutations, all the six genes had a dN/dS rate less than 1, with a not significant Tajima's D and Fu and Li's F neutrality test, thus suggesting that they are under purifying selection (Nei et al., 2010). A MLST scheme with three selected theoretical housekeeping genes (gyrB, toxR, and ompU) has already been used to study clinical and environmental PDD isolated in Japan (Takahashi et al., 2008). However, the authors determined a limit of this MLST approach, that lacked precision when studying the whole population, since genotypic and phenotypic results were not fully congruent. One of the reasons could be that the ompU gene used by these authors could not be a housekeeping gene, since we were not able to detect/amplify it in all our isolates, although a forward primer in a consensus region of the protein was designed (Supplementary Table 2). More recently, the toxR gene alone was used for inferring the probable origin of the Black Sea population of PDD associated to sea bass farms, uncovering a great diversity of the isolates (Terceti et al., 2016).

The improved MLST scheme proposed here allowed the identification of a group of genetically closely related isolates, designated as CC45, with all the isolates included in the subcluster I.b (Figures 1, 2). Most of these isolates (75%) were from cetaceans stranded during an UME period occurred along the Tyrrenian Sea coast of Italy in January-March 2013 (Figure 2). All the other strains were isolated after this mortality event (Supplementary Table 1), with the exception of a SH isolate from a bottlenose dolphin stranded in March 2012. The high genetic relatedness in this group of isolates was confirmed when analyzing the concatenated sequences using different methods, since CC45 isolates always segregated in a separate subcluster.

An intermediate/low recombination level was obtained through the r/m ratio, which is lower than the same ratio observed in other populations of marine bacteria like *Vibrio vulnificus* (Vos and Didelot, 2009). Moreover, the I_A_ value indicates a linkage disequilibrium, compatible with a bacterial population not undergoing relevant recombination events, or with a subpopulation over-represented with respect to the expected value.

These results support the hypothesis that in the Tyrrhenian Sea a specific genetic lineage of PDD (CC45) may have spread among cetaceans (mainly striped dolphins) in the same period of the UME, persisting along the 3-month period of the epidemic outbreak, and apparently beyond. This hypothesis is also supported by the observation of a high intraspecific variability among the other field isolates and the ATCC reference strains, originating from different time and space settings, or from different hosts (e.g., fish, turtles).

Regarding the haemolytic activity, we observed different haemolysis patterns among isolates belonging to different STs, and although a considerable number of haemolytic isolates was found in STs belonging to CC45, the association between the LH or MH phenotypes and CC45 isolates was not significant (Mantel-Haenszel chi-square *p* = 0.30). The phenotypic variability between isolates possessing the same housekeeping genes might suggest the involvement of extra-chromosomal genetic elements, possibility exchanged between different strains, a phenomenon already described among the main evolutionary mechanisms within the *Vibrionaceae* family (Hazen et al., 2010).

From previous studies mostly based on fish isolates, it is already known that the *dly* and *hly*_pl_ genes, harbored in the pPHDD1 plasmid (Rivas et al., 2011), and the *hly*_cr_ gene, harbored in the chromosome (Rivas et al., 2013b), can act synergistically, enhancing the haemolytic effect (Rivas et al., 2013b). Based on this knowledge, we expected the isolates classified as NH to be negative for all the investigated genes, the SH isolates to have only the *hly*_cr_, and the LH and MH to have the *hly*_cr_, together with *dly* and/or *hly*_pl_. In our case, this hypothesis was basically confirmed for the SH and NH field isolates, since all but one of the 19 SH isolates were positive only for *hly*_cr_, with the remaining one being negative for all the haemolysin coding genes and with only one out of the 15 NH field isolates positive for *hly*_cr_, which has been considered a pseudogene (Rivas et al., 2014). On the other hand, 50.3% of the LH field isolates and 13.6% of the MH field isolates were negative for the three considered genes. In general, *hly*_cr_ was most prevalent in SH and MH isolates, while *dly* in LH isolates, with *hly*_pl_ being only rarely detected.

In this regard, it is interesting to note that one of the three sequenced *dly* amplicons showed about 10% differences with the reference *dly*, suggesting the possible existence of different *dly* genes. The existence of other types of haemolysin proteins in the LH/MH isolates studied also cannot be ruled out. After all, the variability of haemolysins in the *Vibrionaceae* is well known (Zhang and Austin, 2005). Moreover, when attempting to visualize plasmids on gel, we found that of the 17 selected isolates only a MH *dly*+, *hly*_*pl*_+, *hly*_*cr*_+ isolate presented a plasmid of the approximate size of the pPHDD1 plasmid. Indeed, other isolates presented more than one plasmid, with no apparent relationship with the haemolytic phenotype (Table 1). The differences in size of the plasmids observed in this study might be explained by the existence of a common core backbone in PDD (Nonaka et al., 2015), which has the capacity of acquiring mobile genetic elements (MGEs) (Dahlberg et al., 1997; Sobecky et al., 1997). In this regard, the phenotypic and genotypic variability observed within PDD isolates might be related to a high permeability of this bacterium to MGEs, including plasmids, but without the occurrence of recombination at the core genome level.

Interestingly, when comparing the proportions of NH, SH, MH and LH isolates detected during the UME period vs. those detected during non-UME periods, isolates with the LH or MH phenotypes were significantly associated (Supplementary Table 3) with the “epidemic event” (UME), and this is also true considering LH and MH isolates altogether vs. SH isolates. More interestingly, our study indicates a possible ecological association between CC45 PDD and the UME period. In other words, the odds of detecting a PDD belonging to CC45 during the UME period was nearly three times greater than detecting a non-CC45 PDD.

Indeed, 57.1% of the isolates in the subcluster I.b presented the LH or MH phenotypes, even though a concordance between the phenotype and the expected virulence gene content was not found, since over 50% of the LH, and over 13% of the MH field isolates did not harbor any of the investigated haemolysin coding genes. Conversely, the virulence gene content of the isolates outside subcluster I.b. was generally consistent with the observed phenotype (Figure 2; Supplementary Table 1). Despite these discrepancies, it has to be underlined that this is the first study reporting the presence of haemolytic genes located on MGEs in PDD from cetaceans. These genes had been already confirmed to play a role in the virulence of PDD in fish and in mice under experimental conditions (Rivas et al., 2011).

The MLST and the molecular characterization of isolates retrieved from marine mammals and reptiles stranded along the Tyrrhenian coasts of Italy could contribute to better understand the population structure of PDD, which is one among the etiologic agents tentatively associated with the UME that occurred in early 2013. In that UME, PDD was isolated from 62% of the cetaceans investigated (Casalone et al., 2014), and our results now indicate that a haemolytic PDD lineage, termed CC45, probably spread within the striped dolphin population residing in the Tyrrhenian Sea in that period.

PDD is known as an important pathogen of fish and mammals (Rivas et al., 2013a), including cetaceans (Fujioka et al., 1988; Buck et al., 1991, 2006), although its role as a major agent of epidemics in marine cetaceans has not yet been demonstrated.

Still, the features obtained by the molecular epidemiology approach we implemented are compatible with an epidemic transmission, irrespective of the role of PDD as a major, or as an opportunistic pathogen causing secondary infections.

In this regard, the MLST scheme proposed in this study showed the capability of clustering isolates detected from hosts with closely related taxonomic rank (e.g., Cetacea) in a short time period and in a defined geographic area, and of segregating this group from: a) other “cetacean isolates” detected in different time periods within the same area; (b) other isolates detected from organisms of different taxonomic ranks (e.g., Fish, Reptiles), and/or originating from other geographic areas (e.g., reference strains). Due to these characteristics, this MLST could represent a useful tool to investigate the population structure or reconstructing the phylogenetic relationships of PDD isolates.

## Author contributions

Conceived and designed the experiments: PA, AC, AF, and AB. Performed the experiments: PA, AI, VD, and LS. Analyzed the data: PA, AC, AF, and AB. Contributed reagents/materials/isolates: AF, AC, CC, FS, GT, GF, and FD. Wrote the paper: PA, AC, AF, and AB.

### Conflict of interest statement

The authors declare that the research was conducted in the absence of any commercial or financial relationships that could be construed as a potential conflict of interest.
