# Supplementary material for: A New Multilocus Sequence Typing Scheme and Its Application for the Characterization of Photobacterium damselae subsp. damselae Associated with Mortality in Cetaceans
Source: Front Microbiol. 2016 Oct 21;7:1656. doi: 10.3389/fmicb.2016.01656 (PMC5073098; doi:10.3389/fmicb.2016.01656)
Supplement: Supplementary file 4 [file Image1.PDF]

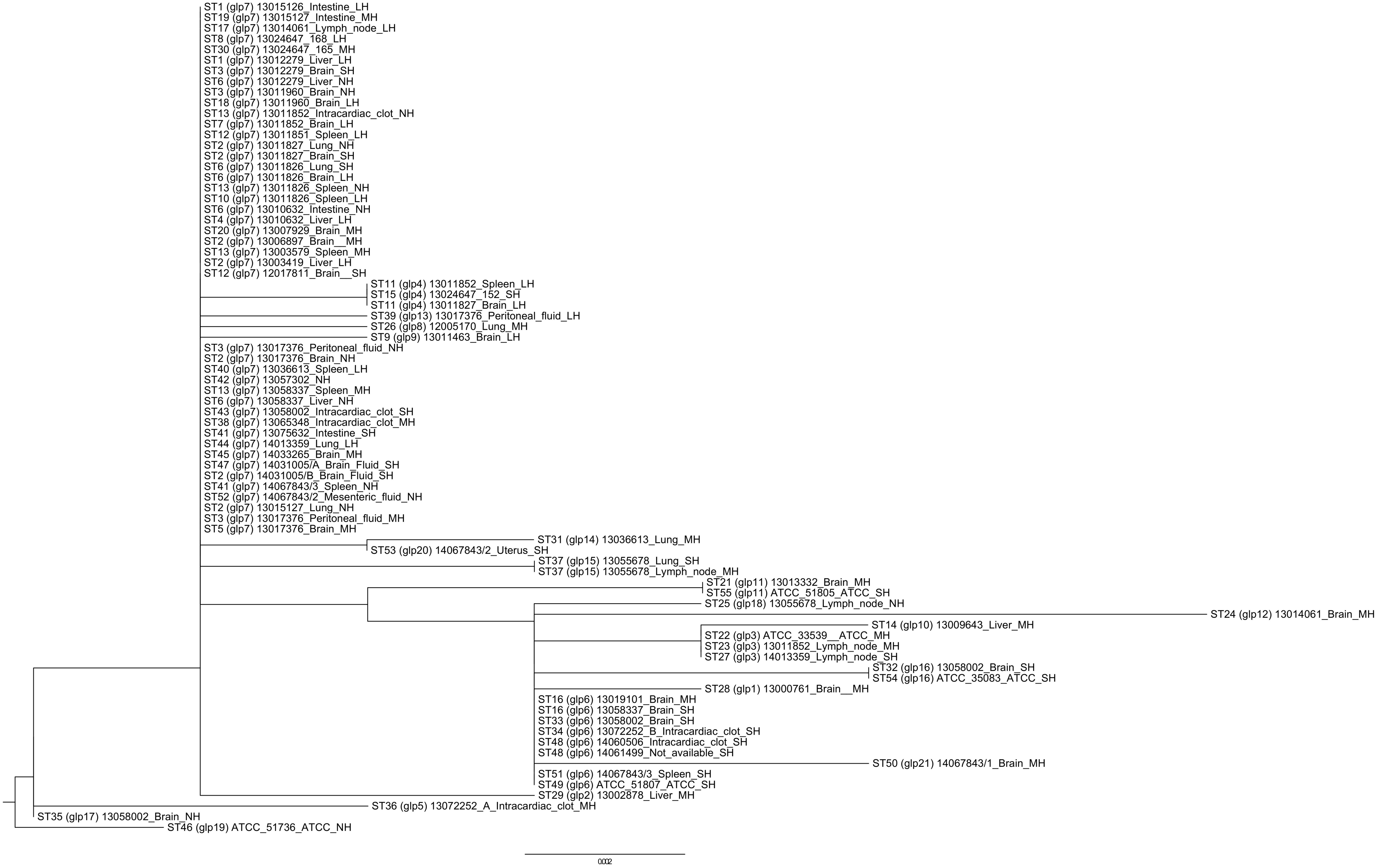

Supplementary figure 1. Phylogenetic tree of the *glp* gene, constructed using Maximum Likelihood.
