# Supplementary material for: A New Multilocus Sequence Typing Scheme and Its Application for the Characterization of Photobacterium damselae subsp. damselae Associated with Mortality in Cetaceans
Source: Front Microbiol. 2016 Oct 21;7:1656. doi: 10.3389/fmicb.2016.01656 (PMC5073098; doi:10.3389/fmicb.2016.01656)
Supplement: Supplementary file 7 [file Image4.PDF]

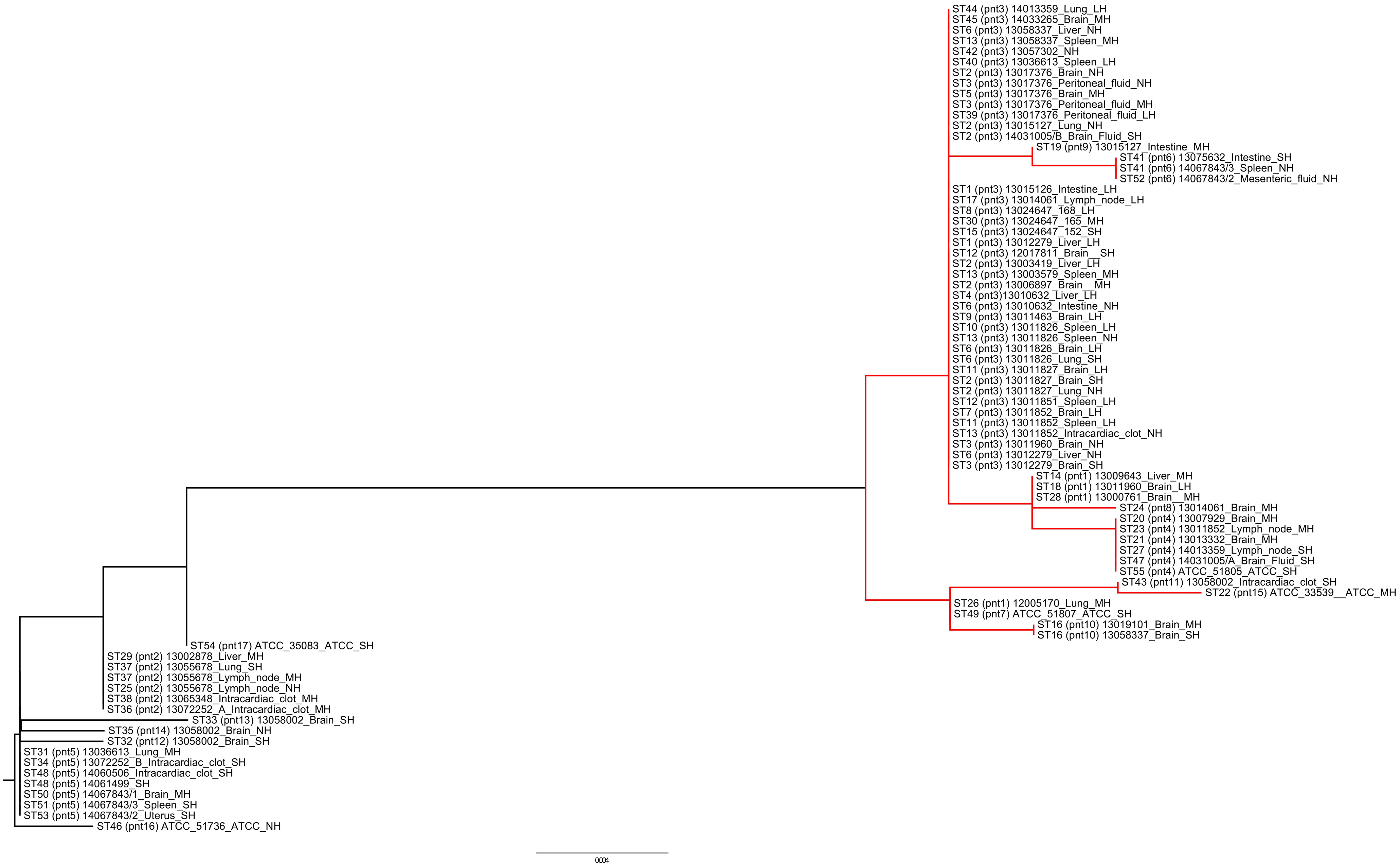

Supplementary figure 4. Phylogenetic tree of the *pnt* gene, constructed using Maximum Likelihood. The red clusters indicate a bootstrap value > 75.
