# Supplementary material for: A New Multilocus Sequence Typing Scheme and Its Application for the Characterization of Photobacterium damselae subsp. damselae Associated with Mortality in Cetaceans
Source: Front Microbiol. 2016 Oct 21;7:1656. doi: 10.3389/fmicb.2016.01656 (PMC5073098; doi:10.3389/fmicb.2016.01656)
Supplement: Supplementary file 11 [file Image8.PDF]

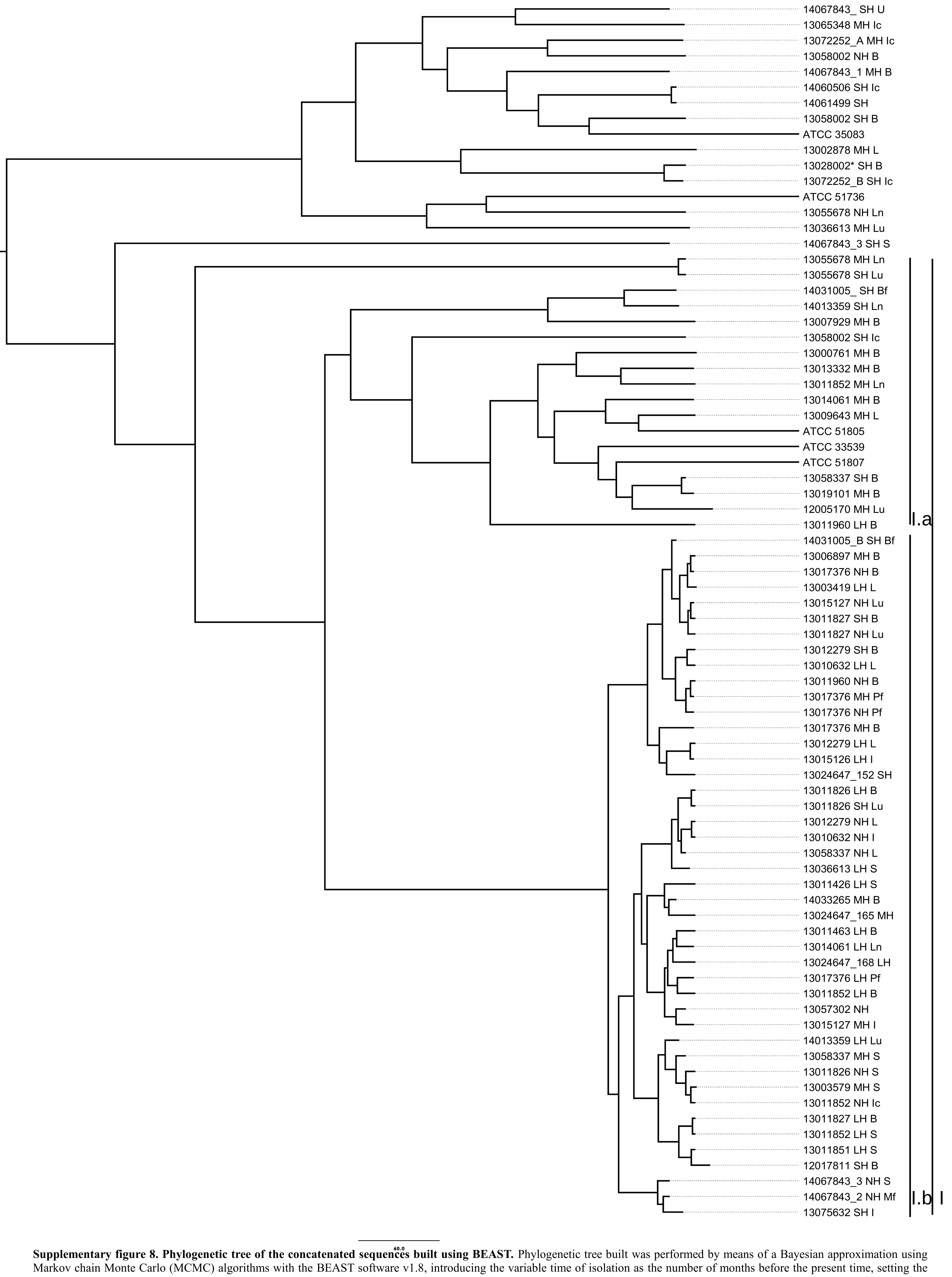

Supplementary figure 8. Phylogenetic tree of the concatenated sequences built using BEAST. Phylogenetic tree built was performed by means of a Bayesian approximation using Markov chain Monte Carlo (MCMC) algorithms with the BEAST software v1.8, introducing the variable time of isolation as the number of months before the present time, setting the present time at January 2015 and hypothesizing a normal clock mutation rate. The ATCC strains were set at 100 months. A total of 1001 trees were obtained. The tree with the highest probability of being the correct one was chosen using TreeAnotator v1.8 (Drummond et al., 2012). LH: large haemolysis; MH: medium haemolysis; SH: small haemolysis; NH: no-haemolysis. B: Brain; I: Intestine; Ic: Intracardiac clot; L: Liver; Ln: Lymph node; Lu: Lung; Mf: Mesenteric fluid; Pf: Peritoneal fluid; S: Spleen; U: Uterus. (13058002 SH B and 13058002* SH B have different STs).
